# Three New Species and One New Record of *Termitomyces* from China

**DOI:** 10.3390/jof12060385

**Published:** 2026-05-27

**Authors:** Wen-Jing Wu, Jin-Chao Yang, Hong-Lin Zhang, Qian Xu, Guang-Rong Zhou, Bo Xu, Yan-Chun Li

**Affiliations:** 1 College of Biological Science and Food Engineering, Southwest Forestry University, Kunming 650201, China; wuwenjing@mail.kib.ac.cn (W.-J.W.); alpine_flora@swfu.edu.cn (B.X.); 2 State Key Laboratory of Phytochemistry and Natural Medicines, Kunming Institute of Botany, Chinese Academy of Sciences, Kunming 650201, China; 3 Yunnan Key Laboratory for Fungal Diversity and Green Development, Kunming Institute of Botany, Chinese Academy of Sciences, Kunming 650201, China; 4 Southwest Survey and Planning Institute of National Forestry and Grassland Administration, Kunming 650031, China; jinchaoyang2026@163.com (J.-C.Y.); 18288967032@163.com (H.-L.Z.); 5 Administrative Bureau of Damingshan Nature Reserve of Guangxi, Nanning 530114, China; xuqian9762@163.com (Q.X.); zgr19317234499@163.com (G.-R.Z.)

**Keywords:** morphology, multi-locus phylogeny, termite mushrooms, new taxa

## Abstract

*Termitomyces* is an economically and ecologically important genus mainly distributed in the southwestern and southern regions of China. We performed phylogenetic analyses of this genus using Maximum Likelihood and Bayesian analyses based on the nuclear ribosomal internal transcribed spacer (ITS), the small subunit of mitochondrial DNA (mtSSU), and the large subunit of nuclear ribosomal DNA (nrLSU) in this study. Three new species, namely *T. albus*, *T. apicoannulatus*, and *T. pseudointermedius*, and one new record to China, *T. pakistanensis*, were described and illustrated based on morphological and molecular evidence. Detailed morphological descriptions, colour photographs, line drawings of microstructures, and comparisons with phylogenetically and morphologically related species are provided.

## 1. Introduction

The genus *Termitomyces* R. Heim was originally established based on the species *T. striatus* (Beeli) R. Heim [1]. Members of this genus are distinguished by several consistent morphological features: a glabrous pileal surface, typically with a pointed perforatorium; white and free lamellae; a white to pale pink spore deposit; a slender or sometimes obtuse stipe that invariably bears a long, root-like pseudorhiza; and typically clavate, obovoid to pyriform, sometimes utriform cheilo- and pleurocystidia [2,3,4,5,6]. As a monophyletic group within the family Lyophyllaceae (Agaricales, Basidiomycota), *Termitomyces* forms a natural evolutionary lineage united by a unique suite of anatomical characteristics and obligate symbiotic habits. All species of this genus form obligate symbiotic associations with fungus-growing termites of the subfamily Macrotermitinae [1,3,7,8,9,10]. This mutualistic association, in which the fungus forms the essential fungal crop in termite-cultivated fungus combs, underpins the ecological success and broad geographic distribution of the genus. Species of this genus are largely confined to tropical and subtropical regions, with major diversity hotspots in Africa and Asia [7,8]. Beyond their ecological role, many species are highly valued edible mushrooms and constitute a significant economic component of local markets and subsistence economies across their distribution range [11,12,13,14,15,16,17,18,19,20,21,22,23,24,25,26].

Early efforts to classify Chinese *Termitomyces* led to the proposal of a separate genus, *Sinotermitomyces* M. Zang [27]. However, subsequent molecular phylogenetic works [3,19,20,21,22,23,24,25,26], notably by Frøslev et al. [3], indicated that this taxon is best synonymized under *Termitomyces*. Following this unification, Wei et al. [28,29] described a new species, *T. bulborhizus* T.Z. Wei, Y.J. Yao, Bo Wang & Pegler, and recognized eleven species in China based on morphological analyses. Further contributions included the report of *T. intermedius* as a new record for China [20]. In recent years, the application of integrated morphological and molecular phylogenetic approaches has yielded several new species and new records from China [4,6,16,26,30,31]. Worldwide, 71 species have been reported in the genus *Termitomyces* (Index Fungorum, https://www.indexfungorum.org/Names/Names.asp, accessed on 10 April 2026).

Despite increased taxonomic efforts both globally and regionally, the diversity and systematics of *Termitomyces* in China remain inadequately documented. Many putative new taxa still lack formal description, and comprehensive phylogenetic frameworks that include recent collections are needed. The present study documents three new species and one new record of *Termitomyces* from China based on morphological characteristics and molecular phylogenetic evidence using the internal transcribed spacer (ITS), the mitochondrial small subunit (mtSSU), and the nuclear ribosomal large subunit (nrLSU).

## 2. Materials and Methods

### 2.1. Specimen Collections

Specimens of *Termitomyces* were gathered across China’s Yunnan and Guangxi Provinces. Fresh basidiomata were photographed in the field, and macroscopic features were documented for each specimen, accompanied by field notes including the collection date, geographic location, and habitat details. Specimens were dried at 45 °C using a food dehydrator, then stored in sealed plastic bags and deposited in the fungal herbarium (HKAS) of the Herbarium KUN (Kunming Institute of Botany, Chinese Academy of Sciences) for further taxonomic research. Additionally, small samples from each specimen were preserved in silica gel for subsequent molecular analyses.

### 2.2. Morphological Studies

Macroscopic features were obtained via specimen data sheets and field photographs, following the colour codes defined by Kornerup and Wanscher [32]. For microscopic features, the structures of the pileipellis, context hyphae, subhymenium hyphae, basidia, cystidia, and basidiospores were examined using a ZEISS Axiostar Plus microscope (Carl Zeiss AG, Oberkochen, Germany). Tissues were sectioned and mounted in 10% KOH for rehydration. The sections were then stained with Cotton Blue to test for cyanophily and Melzer’s reagent to test for amyloidity and dextrinoidity [23,33,34]. Microscopic illustrations of the basidiospores, pileipellis, and hymenium were drawn freehand under 1000× magnification. At least 20 basidiospores from each basidioma were measured, with the notation “basidiospores (n/m/p)” indicating n basidiospores measured from m basidiomata of p specimens. Spore length (L) and width (W) represent the arithmetic averages of all measured spores, while Q refers to the variation in the length/width ratios of basidiospores in side view. Q_m_ indicates the average Q ± sample standard deviation [35,36,37]. Since the shape of basidiospores is largely determined by their length/width ratio, the following categories are defined: globose (L/W ratio = 1.01–1.05), subglobose to broadly ellipsoid (L/W ratio = 1.05–1.3), ellipsoid (L/W ratio = 1.3–1.6), and elongated (L/W ratio = 1.6–2). The term “masl” refers to the height above sea level [38].

### 2.3. DNA Extraction, PCR, and DNA Sequencing

Genomic DNA was extracted from dried specimens using the CTAB method [39]. The PCR mixture consisted of 1 μL of DNA solution (approximately 20 ng), 1 μL of each primer, and 15 μL of 2 × Taq PCR Master Mix, containing Taq DNA Polymerase, MgCl_2_, and dNTPs (Beijing Biomed Gene Technology Co., Ltd., Beijing, China). The final volume was adjusted to 30 μL with distilled sterile water. The PCR conditions were as follows: denaturation at 95 °C for 4 min, followed by 35 cycles of 30 s at 94 °C, 40 s at 53 °C, and 1 min at 72 °C, with a final extension at 72 °C for 8 min and a cooling step at 14 °C [40,41]. In this study, the primers used for nrLSU amplification were LR0R and LR5 [42]; the mtSSU region was amplified with *Termitomyces*-specific primer pairs, viz., SSUFW105 and SSUREV475 [43]; and the internal transcribed spacer (ITS) regions were amplified using primers ITS1F/ITS4 [34,44,45,46]. When full ITS region amplification failed, the sequence was divided into two overlapping fragments and successfully amplified using primer pairs ITS1F/5.8S and 5.8SR/ITS4 [43]; the sequences from both fragments were then concatenated to generate the complete ITS sequence. Newly generated sequences were deposited in GenBank (Table 1).

### 2.4. Sequence Alignment and Phylogenetic Analyses

DNA sequences were assembled using SeqMan (DNASTAR Lasergene v.9). Sequences of *Termitomyces* generated in this study and selected sequences retrieved from GenBank (Table 1) were aligned using Mafft V7.490 [53] and further refined manually with PhyDE version 0.9971 [54] where necessary.

Phylogenies and node support values were first inferred by Maximum Likelihood (ML) from the three single-locus datasets separately, using RAxMLGUI 2.0.10 [55] with the GTRGAMMAI model and 1000 bootstrap replicates, in combination with an ML search. Since no conflicts (BS ≥ 70%) were detected among the topologies, the three single-locus datasets were concatenated using Sequence Matrix [56]. Partitioned ML analysis was performed on the concatenated dataset, as described above. The Bayesian inference (BI) analysis was performed with MrBayes 3.2 [57]. The best-fit model for the ITS-mtSSU-nrLSU dataset was GTR + F + G4+ I, determined by the Akaike Information Criterion (AIC) and ModelFinder (PhyloSuite version 1.2.3) [58,59,60]. Two runs of six chains each and sampled every 1000 generations were stopped after 9,065,000 generations, when the average standard deviation of split frequencies went below 0.01. The first 25% of the generations were discarded as burn-in, and Bayesian PPs were then calculated from the posterior distribution of the retained Bayesian trees. A clade was considered to be strongly supported if it showed a bootstrap support value (BS) ≥ 70% and a posterior probability (PP) ≥ 0.90. Phylogenetic trees were displayed in FigTree v. 1.4.0 (https://tree.bio.ed.ac.uk/software/figtree/, accessed on 6 February 2026). Genetic distances and sequence alignment statistics for each gene fragment and the concatenated dataset were calculated using the p-distance model in MEGA 12 [61].

## 3. Results

### 3.1. Phylogenetic Analyses

**Figure 1:**
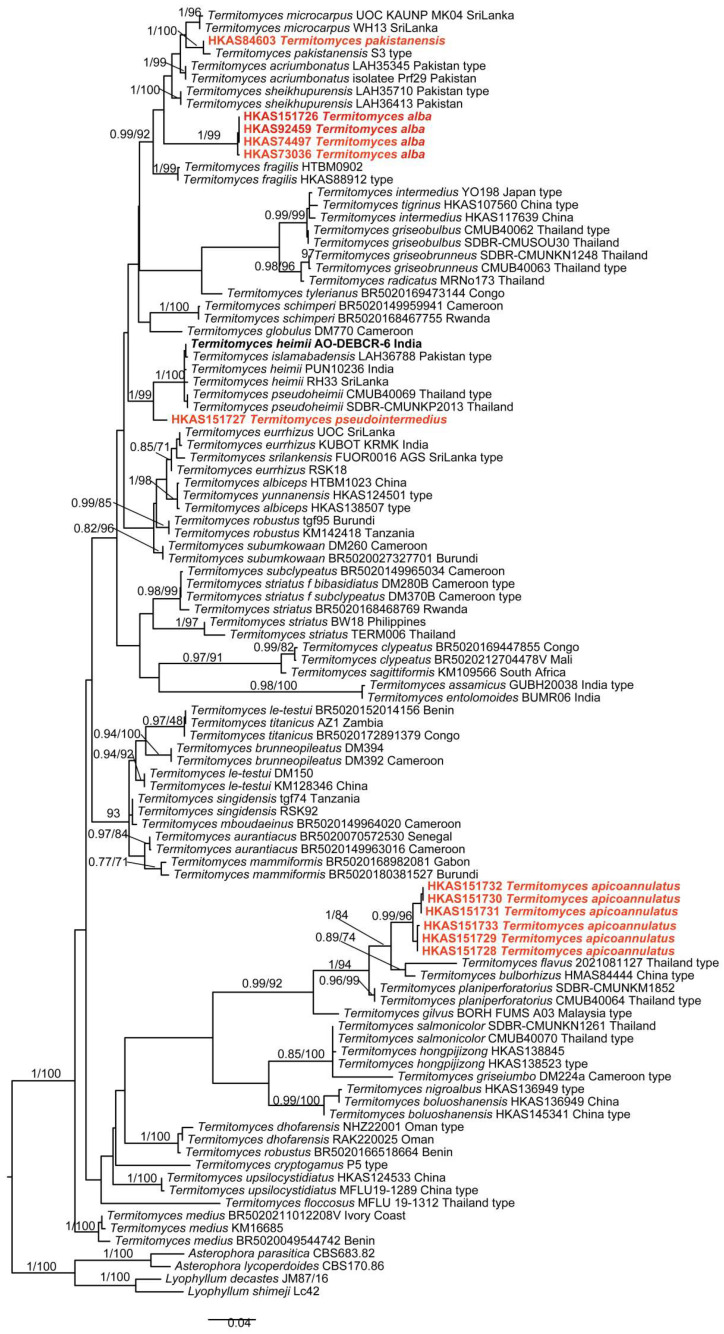
Phylogenetic relationships among species of *Termitomyces* inferred from combined ITS, mtSSU and nrLSU sequences, using the Maximum Likelihood and Bayesian inference approaches (ML topology is shown). The ML bootstrap support (≥50) and Bayesian posterior probability (≥0.95) are shown at the branches (PP/BS). Sequences newly generated in this study are in red. The bold sequence marks the representative species of this clade. Vouchers are indicated after the species names. *Lyophyllum shimeji*, *L. decastes*, *Asterophora lycoperdoides* and *A. parasitica* were used as outgroup taxa.

Phylogenetic analyses were conducted based on 80 ITS sequences, 62 nrLSU sequences and 47 mtSSU sequences, of which 12 ITS sequences, nine nrLSU sequences and nine mtSSU sequences were newly generated in this study (Table 1). *Lyophyllum shimeji* (Kawam.) Hongo, *L. decastes* (Fr.) Singer, *Asterophora lycoperdoides* (Bull.) Ditmar, and *A. parasitica* (Bull.) Singer were used as the outgroup taxa [6]. The concatenated dataset consisted of 2657 bp, including gaps, and contained 704 parsimony-informative sites. The breakdown by individual marker was as follows: the ITS fragment was 1457 bp with 403 parsimony-informative sites; the nrLSU fragment measured 821 bp with 236 parsimony-informative sites; and the mtSSU fragment was 379 bp with 82 parsimony-informative sites. The combined alignment was submitted to TreeBASE (S30371). ML and BI approaches showed minimal differences in their evaluation results; thus, only the ML tree was used for display (Figure 1). Our phylogenetic analyses indicated that sequences of the new species *T. albus* form a distinct lineage with high support values (1/100) and cluster together with *T. acriumbonatus* Usman & Khalid, *T. sheikhupurensis* Izhar, Khalid & H. Bashir, *T. microcarpus* (Berk. & Broome) R. Heim and *T. pakistanensis* A. Razaq. The sequence of the new species *T. pseudointermedius* clusters together with those of *T. heimii* Natarajan *T. islamabadensis* S. Ashraf, Usman & Khalid, and *T. pseudoheimii* Paloi, Suwannar. & Kuml with high support values (1/99). The new species *T. apicoannulatus* is closely related and clusters together with *T. flavus* S.M. Tang & S.H. Li and *T. bulborhizus* T.Z. Wei, Y.J. Yao, Bo Wang & Pegler with high support values (1/84).

### 3.2. Taxonomy

**Figure 2:**
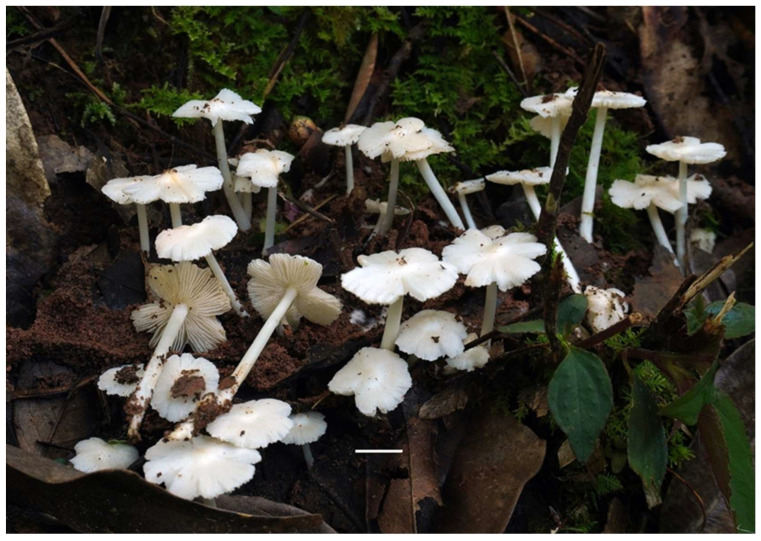
Fresh basidiomata of *Termitomyces albus*. Bar = 10 mm (KUN-HKAS151726, holotype).

**Figure 3:**
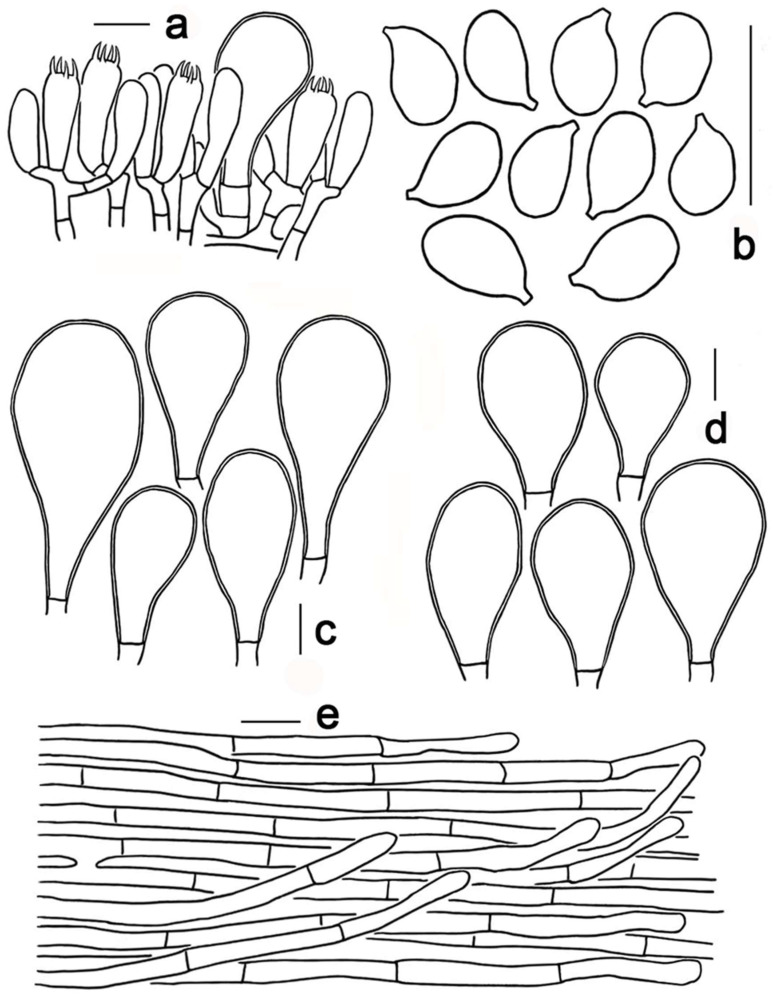
*Termitomyces albus* (KUN-HKAS151726, holotype). (**a**) Basidia, basidioles and pleurocystidium; (**b**) basidiospores; (**c**) cheilocystidia; (**d**) pleurocystidia; (**e**) pileipellis. Scale bars: (**a**–**e**) = 10 µm.

*Termitomyces albus* Yan C. Li, Wen J. Wu, sp. nov. (Figure 2 and Figure 3)

MycoBank: 862706.

Etymology: “*Albus*” refers to the white basidiomata of this species.

Type: China, Yunnan Province: Pu’er, Sun-River National Park, 22.59°N, 101.11°E, altitude 1430 masl, 20 September 2025, Wen-Jing Wu 018 (KUN-HKAS151726, GenBank Acc. No. ITS: PX924552, nrLSU: PX916413, mtSSU: PX924563).

Diagnosis: *Termitomyces albus* is distinguished from other species of this genus by its small, entirely white basidiomata; applanate pileus with a blunt perforatorium; fistulose, frangible stipe; elongated basidiospores; and thick-walled cheilo- and pleurocystidia.

Description: Basidiomata very small to small. Pileus 0.8–2.5 cm in diam., applanate, with a blunt perforatorium at centre; surface white (1A1), margin more or less radially splitting; context white (1A1), unchanging in colour when bruised. Lamellae free, dense, white (1A1), with small lamellulae, margin serrate. Stipe 2–4.3 × 0.1–0.25 cm, central, cylindrical, white (1A1), surface smooth, fistulose and frangible; context white, fibrous. Annulus absent. Pseudorhiza absent or present; when present, with a ceramic white (1A1) surface, connected to subterranean termite nests. Odour indistinct.

Basidia 18–26 × 7–10 μm, clavate, mostly four-spored, rarely two-spored, thin-walled, nearly hyaline in 10% KOH. Basidiospores [200/10/4] 6–7.5 (–8) × 3–4.5 (–5) μm, sterigmata 2–4 μm long, Lm × Wm = 6.8 ± 0.5 × 3.9 ± 0.4 μm, Q = 1.6–1.8, Q_m_ = 1.74 ± 0.22, elongated, thin-walled, smooth, nearly hyaline in 10% KOH, non-amyloid, non-dextrinoid. Cheilocystidia abundant, 28–60 × 17–28 μm, thick-walled (1–2 μm thick), clavate, utriform or pyriform, nearly hyaline in KOH, scattered. Pleurocystidia abundant, 20–42 × 17–26 μm, similar to cheilocystidia in shape, thick-walled (1–2 μm thick), nearly hyaline in KOH. Pileipellis a cutis, composed of radially arranged interwoven to subparallel hyphae; terminal cells 18–47 × 2–5 μm, clavate to cylindrical. Clamp connections absent in all tissues.

Habitat: Gregarious on the ground above the subterranean termitarium.

Distribution: Currently known from Yunnan Province, China.

Additional specimens examined: China, Yunnan Province: Wenshan Prefecture, Qiubei County, Zhongxiang Village, 8 August 2015, Zhao Kuan 829 (KUN-HKAS92459, GenBank Acc. No. ITS: PX924555, nrLSU: PX916414, mtSSU: PX924564); Nujiang Lisu Autonomous Prefecture, Lushui City, Laowo Town, Chongren Village, 7 August 2011, Yuan Mingsheng 1017 (KUN-HKAS74497, GenBank Acc. No. ITS: PX924554); Dali Bai Autonomous Prefecture, Binchuan County, Jizushan Town, Nanshan, 1 September 2011, Yuan Mingsheng BC-74 (KUN-HKAS73036, GenBank Acc. No. ITS: PX924553, mtSSU: PX924565).

Notes: *Termitomyces albus* is characterized by its small, entirely white basidiomata; applanate pileus with a blunt central perforatorium; white, cylindrical, fistulose, frangible stipe; absent pseudorhiza; elongated basidiospores, and thick-walled cheilo- and pleurocystidia.

The differences between *T. albus* and several related small-sized *Termitomyces* species, i.e., *T. fragilis* L. Ye, Karun., J.C. Xu, K.D. Hyde & P.E. Mortimer, *T. acriumbonatus*, *T. sheikhupurensis*, *T. microcarpus* and *T. pakistanensis*, are as follows: *T. fragilis* has a brownish grey to greyish white pileus which typically exceeds 3 cm in diam., surface mixed with tiny white filamentous striations, and obtusely pointed perforatorium [4]. *Termitomyces acriumbonatus* has a creamy white pileus with slightly greyish striations, a greyish brown pointed perforatorium, subglobose to ellipsoid basidiospores measuring 6.1–8.7 × 4.5–6.5 μm, and distinct pseudorhiza [25]. *Termitomyces microcarpus* has a relatively large pileus (up to 5 cm in diam.) which is pale grey or with a faint ochraceous tinge at the centre and white or cream-coloured towards the margin, a papilla perforatorium, a solid stipe, a white pseudorhiza, ovoid to ellipsoid basidiospores measuring 5.6–6.9 × 3.7–4.8 μm, and thin-walled pleuro- and cheilocystidia [2]. *Termitomyces pakistanensis* has a cream pileus with camel brown fibrillose, olive grey to brown stipe, and ellipsoid to ovoid basidiospores measuring 4.1–8.8 × 3.7–6.1 μm [50]. *Termitomyces sheikhupurensis* has a light brownish grey to dull orange pileus with a conspicuous nipple-shaped perforatorium, surface radially mixed with white sulcate striations; a pale yellow to light brownish grey solid stipe; subelliptic to ellipsoid basidiospores measuring 5.5–8.07 × 4.4–6.13 μm; and thin-walled, polymorphic cheilo- and pleurocystidia [5]. The interspecific genetic distances between *T. albus* and the above species are as follows: *T. acriumbonatus* (0.116), *T. sheikhupurensis* (0.109), *T. microcarpus* (0.099), *T. pakistanensis* (0.138), and *T. fragilis* (0.129).

**Figure 4:**
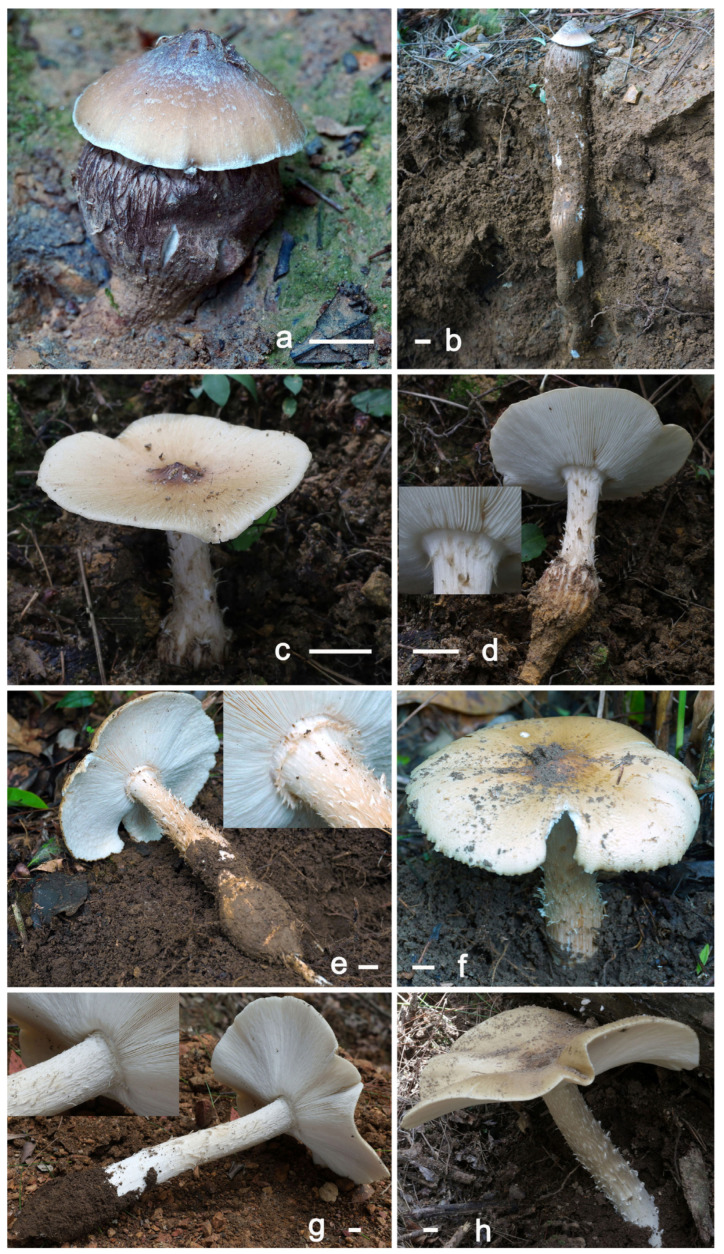
Fresh basidiomata of *Termitomyces apicoannulatus*. Bars = 10 mm. ((**a**,**b**): HKAS151728; (**c**,**d**): KUN-HKAS151729; (**e**,**f**): KUN-HKAS151733, holotype; (**g**,**h**): KUN-HKAS151730).

**Figure 5:**
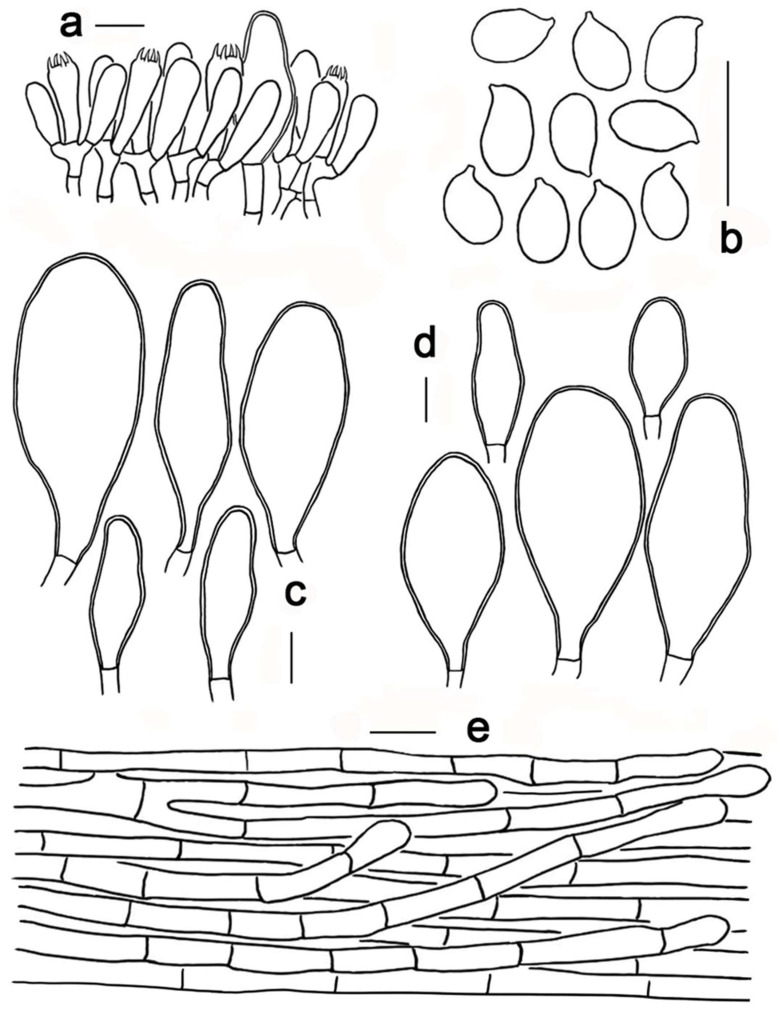
*Termitomyces apicoannulatus* (KUN-HKAS151733, holotype). (**a**) Basidia, basidioles and pleurocystidium; (**b**) basidiospores; (**c**) cheilocystidia; (**d**) pleurocystidia; (**e**) pileipellis. Scale bars: (**a**–**e**) = 10 µm.

*Termitomyces apicoannulatus* Yan C. Li, Wen J. Wu, sp. nov. (Figure 4 and Figure 5)

MycoBank: 862708.

Etymology: “*Apicoannulatus*” highlights the key diagnostic feature: the apical annulus.

Type: China, Guangxi Province: Nanning City, Shanglin County, Xiangxian Town, Liulian Village, Guangxi Daming Mountain National Nature Reserve, 23.29 N, 108.60 E, altitude 230 masl, 7 August 2025, Yan-Chun Li 8384 (KUN-HKAS151733, GenBank Acc. No. ITS: PX924559, nrLSU: PX916418, mtSSU: PX924571).

Diagnosis: *Termitomyces apicoannulatus* is distinguished from other species of this genus by its brownish yellow to orange-white pileus always with a blunt perforatorium, the glabrous pileal surface always covered with detachable white tomentum when young; a pale orange stipe covered with white fibrillose scales; the presence of an apical, often evanescent annulus; a brown pseudorhiza; elongated basidiospores; and thick-walled cheilo- and pleurocystidia.

Description: Basidiomata small to medium-sized. Pileus 3.2–23.4 cm in diam., conical, covered with easily detachable white tomentum when young; becoming applanate at maturity with a blunt perforatorium at centre; surface brownish orange or brownish yellow (5C6–5C8) to light brown or dark brown (6D8–6F8) at centre, remaining parts greyish orange, light brown, orange-white to yellowish white (6B4–6D6, 5A2–5B4, 2A2–4A2), gradually paler towards margin; margin involute; context white (1A1), unchanging in colour when bruised. Lamellae free, dense, white (1A1), with small lamellulae, margin serrate. Stipe 3.3–20.4 × 0.6–2.5 cm, cylindrical, orange-white to pale orange (5A2–5A3), subcylindrical, solid, fibrous, surface densely to sparsely covered with white fibrillose scales, nearly uniform in width or noticeably swollen at base, surface brown (6E8); context white, fibrous. Annulus apical, often fragmented and disappearing when mature. Pseudorhiza present, connected to subterranean termite nests; surface brown (6E8); context solid, fibrous. Odour slightly fragrant.

Basidia 17–22 × 5–7 μm, clavate, mostly four-spored, rarely one-, two- or three-spored, thin-walled, nearly hyaline in 10% KOH. Basidiospores [60/3/3] 6–7 (–8) × 3–4 (–5) μm, sterigmata 2–4 μm, Lm × Wm = 6.8 ± 0.6 × 3.6± 0.6 μm, Q = 1.6–2.0, Q_m_ = 1.89 ± 0.36, elongated, thin-walled, smooth, nearly hyaline in 10% KOH, non-amyloid, non-dextrinoid. Cheilocystidia abundant, 28–65 × 9–25 μm, thick-walled (1–2 μm thick), clavate to utriform, nearly hyaline in KOH, scattered. Pleurocystidia abundant, 25–57 × 10–26 μm, similar to cheilocystidia in shape, thick-walled (1–2 μm thick), nearly hyaline in KOH. Pileipellis a cutis, composed of radially arranged interwoven to subparallel hyphae; terminal cells 13–36 × 3–6 μm, clavate to subcylindrical. Clamp connections absent in all tissues.

Habitat: Solitary on the ground above the subterranean termitarium.

Distribution: Currently known from Guangxi Province, China.

Additional specimens examined: China, Guangxi Province: Nanning City, Shanglin County, Xiangxian Town, Liulian Village, Guangxi Daming Mountain National Nature Reserve, 23.29 N 108.60 E, altitude 230 masl, 7 August 2025, Yan-Chun Li 8381 (KUN-HKAS151731, GenBank Acc. No. ITS: PX924561, nrLSU: PX916420, mtSSU: PX924569); nearby this location, altitude 465 masl, 9 August 2025, Yan-Chun Li 8383 (KUN-HKAS151732, GenBank Acc. No. ITS: PX924562, nrLSU: PX916421, mtSSU: PX924570); Shanglin County, Dafeng Town, Dongchun Village, Guangxi Daming Mountain National Nature Reserve, 23.29 N 108.60 E, altitude 230 masl, 9 August 2025, Yan-Chun Li 8229 (KUN-HKAS151728, GenBank Acc. No. ITS: PX924557, nrLSU: PX916416); nearby this location, the same date, altitude 498 masl, Yan-Chun Li 8296 (KUN-HKAS151729, GenBank Acc. No. ITS: PX924558, nrLSU: PX916417, mtSSU: PX924567); Shanglin County, Mingliang Town, Luozhen Village, Guangxi Daming Mountain National Nature Reserve, 23.29 N 108.60 E, altitude 400 masl, 10 August 2025, Yan-Chun Li 8322 (KUN-HKAS151730, GenBank Acc. No. ITS: PX924560, nrLSU: PX916419, mtSSU: PX924568).

Notes: *Termitomyces apicoannulatus* is characterized by a brownish yellow to orange-white pileus with a blunt perforatorium; a pale orange stipe covered with white fibrillose scales; an apical, often evanescent annulus; a brown pseudorhiza; elongated basidiospores; and thick-walled cheilo- and pleurocystidia.

In our phylogenetic analysis, *T. apicoannulatus* clustered together with *T. bulborhizus* (interspecific genetic distance 0.085), *T. flavus* (interspecific genetic distance 0.310), *T. gilvus* C.S. Yee & Seelan (interspecific genetic distance 0.079) and *T. planiperforatorius* Paloi & Suwannar (interspecific genetic distance 0.064). However, *T. bulborhizus* has a brown to pale brown pileus, whitish or fulvous brown stipe, prominent globose bulbous stipe base, absent annulus, and ovoid to ellipsoid basidiospores measuring 6.0–9.0 × 4.0–6.0 μm [28]. *Termitomyces flavus* has a large pileus that can reach 40 cm wide, a brownish orange to brownish yellow pileal surface, a whitish or fulvous brown stipe, absent annulus, and ellipsoid basidiospores measuring 6.0–8.8 × 4.1–6.2 μm [26]. *Termitomyces gilvus* has a brownish orange pileus, clavate and relatively large basidia (21.8–29.2 × 6.1–8.3 μm), and thin-walled cheilo- and pleurocystidia [24]. *Termitomyces planiperforatorius* has a greyish orange to light brown pileus with a slightly round to flat perforatorium, a cracked pileal surface with filamentous squamules when mature, and thin-walled cheilo- and pleurocystidia [51].

**Figure 6:**
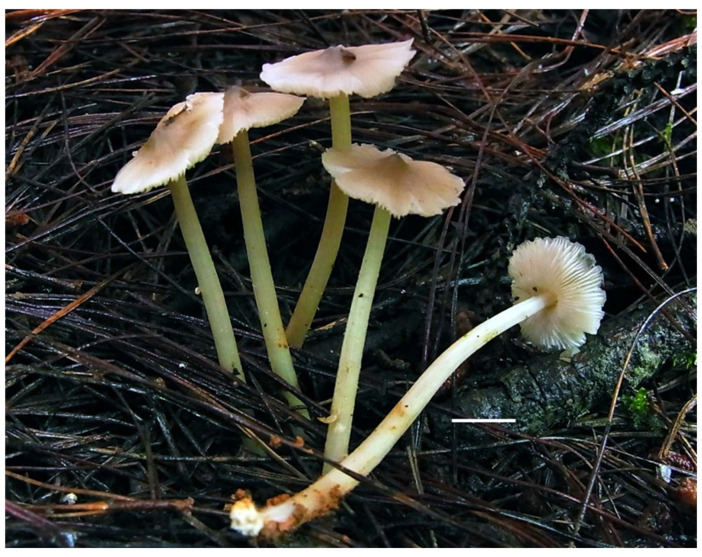
Fresh basidiomata of *Termitomyces pakistanensis*. Bar = 10 mm (KUN-HKAS84603).

**Figure 7:**
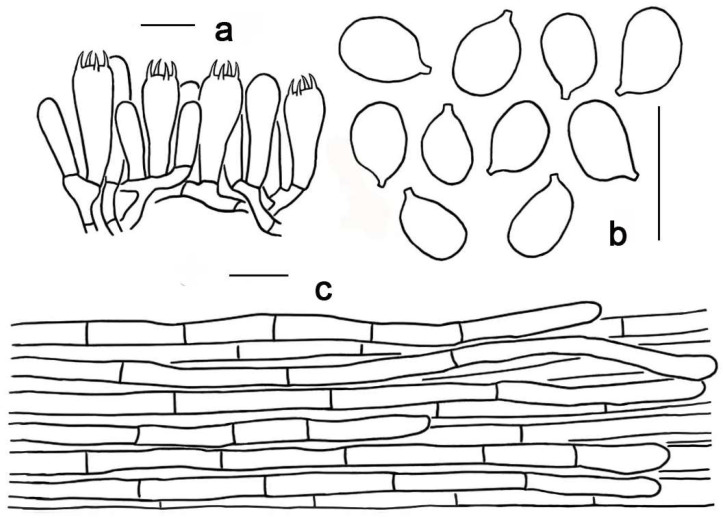
*Termitomyces pakistanensis* (KUN-HKAS84603). (**a**) Basidia, basidioles; (**b**) basidiospores; (**c**) pileipellis. Scale bars: (**a**–**c**) = 10 µm.

*Termitomyces pakistanensis* A. Razaq, in Razaq, Ishaq, Ilyas & Sadia, *Microsc. Res. Tech.* 86(1): 117 (2023). (Figure 6 and Figure 7)

Basidiomata small. Pileus 1–2.4 cm in diam., initially conical to parabolic, becoming applanate at maturity, with a blunt perforatorium at centre; surface olive brown to yellowish brown (4E8, 5E7–5E8), brownish orange to orange-white to white (6C5–6C4, 6A2–6A1) elsewhere, gradually lightening from the centre toward the margin; margin more or less radially splitting; context white (1A1), unchanging in colour when bruised. Lamellae free, moderately spaced, white (1A1), with small lamellulae, margin smooth to serrate. Stipe 5–7.5 × 0.2–0.3 cm, central, cylindrical, white (1A1), surface smooth, fistulose and frangible. Annulus absent. Pseudorhiza absent, connected to subterranean termite nests. Odour indistinct.

Basidia 20–24 × 5–7 μm, clavate, mostly four-spored, rarely one-, two- or three-spored, thin-walled, nearly hyaline in 10% KOH. Basidiospores [120/6/1] 6–7 (–7.5) × 4–5 μm, sterigmata 2–4 μm, Lm × Wm = 6.0 ± 0.5 × 4.3 ± 0.5 μm, Q = 1.3–1.7, Q_m_ = 1.40 ± 0.20, ellipsoid to elongated, thin-walled, smooth, nearly hyaline in 10% KOH, non-amyloid, non-dextrinoid. Cheilo- and pleurocystidia are not observed. Pileipellis a cutis, composed of radially arranged interwoven to subparallel hyphae; terminal cells 18–50 × 4–7 μm, clavate to subcylindrical. Clamp connections absent in all tissues.

Habitat: Gregarious on the ground above the subterranean termitarium.

Distribution: Currently known from Pakistan and China.

Specimen examined: China, Yunnan Province: Baoshan City, Longyang District, Lujiang Town, Bawan Village, Gaoligong Mountain near the Nankang Management Station, 17 June 2014, Li-Hong Han 307 (KUN-HKAS84603, GenBank Acc. No.ITS PX953039).

Notes: The ITS sequence from the Chinese collection is almost identical to that of *T. pakistanensis* from the type specimen (genetic distance 0.007), suggesting that they are conspecific. *Termitomyces pakistanensis* is originally characterized by its cream pileus with camel brown fibrillose squamules; a long, slender, hollow stipe; absent annulus and pseudorhiza; moderately small, ellipsoid to ovoid basidiospores; and clavate cheilo- and pleurocystidia [50]. However, the Chinese specimen has an olive brown to brownish orange pileus, a smooth pileal surface, and an absence of cheilo- and pleurocystidia. These traits supplement the characteristics of this species.

**Figure 8:**
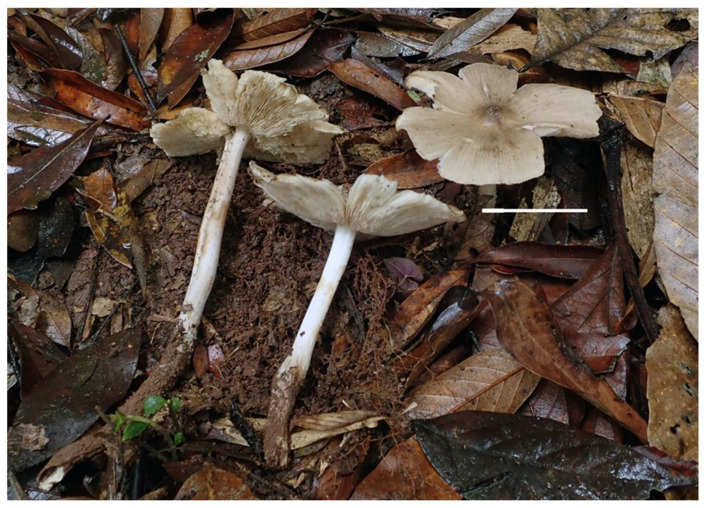
Fresh basidiomata of *Termitomyces pseudointermedius*. Bar = 50 mm (KUN-HKAS151727, holotype).

**Figure 9:**
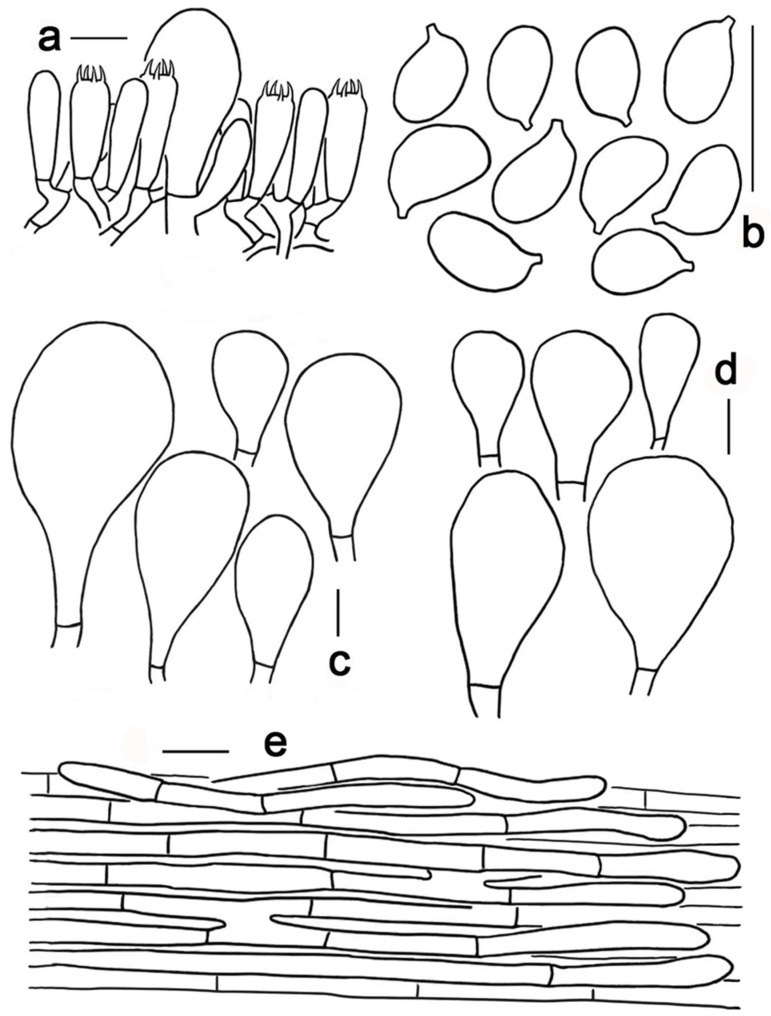
*Termitomyces pseudointermedius* (KUN-HKAS151727, holotype). (**a**) Basidia, basidioles and pleurocystidium; (**b**) basidiospores; (**c**) cheilocystidia; (**d**) pleurocystidia; (**e**) pileipellis. Scale bars: (**a**–**e**) = 10 µm.

*Termitomyces pseudointermedius* Yan C. Li, Wen J. Wu, sp. nov. (Figure 8 and Figure 9)

MycoBank: 862707.

Etymology: “*Pseudointermedius*” refers to this species being morphologically similar to *T. intermedius*.

Type: China, Yunnan Province: Pu’er City, Jiangcheng County, Menglie Town, 22.31° N, 101.53° E, altitude 1047 masl, 30 June 2019, Si-Peng Jian 633 (KUN-HKAS151727, GenBank Acc. No. ITS: PX924556, nrLSU: PX916415, mtSSU: PX924566).

Diagnosis: *Termitomyces pseudointermedius* is distinguished from other species of this genus by a grey to greyish beige pileus, with a blunt and greyish brown to brown perforatorium at the centre, and concolorous sulcate striations towards the margin; a white, solid, smooth stipe; greyish white pseudorhiza; ellipsoid to elongated basidiospores; and thin-walled cheilo- and pleurocystidia.

Description: Basidiomata medium-sized. Pileus 9.5–10.5 cm in diam., applanate, with a blunt perforatorium at centre; surface greyish brown to brown (5E3–5F4) at centre, grey to greyish beige (4B1–4C2) elsewhere, gradually paler towards margin; radially mixed with concolorous sulcate striations, margin more or less radially splitting; context white (1A1), unchanging in colour when bruised. Lamellae free, dense, white (1A1), with small lamellulae, margin smooth to serrate. Stipe 13–19 × 0.8–1 cm, cylindrical, white (1A1), solid, surface nearly glabrous; context white, fibrous. Annulus absent. Pseudorhiza present, connected to subterranean termite nests; greyish white (1B1); context solid, fibrous. Odour slightly fragrant.

Basidia 17–22 × 6–8 μm, clavate, mostly four-spored, rarely one-, two- or three-spored, thin-walled, nearly hyaline in 10% KOH. Basidiospores [60/3/1] (6–) 7–8 × 4–5 μm, sterigmata 2–4 μm, Lm × Wm = 7.5 ± 0.4 × 4.4 ± 0.5 μm, Q = 1.5–1.8, Q_m_ = 1.70 ± 0.21, ellipsoid to elongated, thin-walled, smooth, nearly hyaline in 10% KOH, non-amyloid, non-dextrinoid. Cheilocystidia seldom, 26–68 × 17–32 μm, thick-walled (1–2 μm thick), clavate, utriform or pyriform, nearly hyaline in KOH, scattered. Pleurocystidia abundant, 22–41 × 10–25 μm, similar to cheilocystidia in shape, thick-walled (1–2 μm), nearly hyaline in KOH. Pileipellis a cutis, composed of radially arranged interwoven to subparallel hyphae; terminal cells 18–35 × 4–5 μm, subclavate to subcylindrical. Clamp connections absent in all tissues.

Habitat: Scattered on the ground above the subterranean termitarium.

Distribution: Currently known from Yunnan Province, China.

Notes: *Termitomyces pseudointermedius* is characterized by a greyish beige pileus with a blunt perforatorium at centre and concolorous radially arranged sulcate striations at the margin, a white stipe, a greyish white pseudorhiza, ellipsoid to elongated basidiospores, and thin-walled cheilo- and pleurocystidia.

In our phylogenetic analyses, this species clustered with another clade consisting of three taxa: *T. heimii*, *T. islamabadensis*, and *T. pseudoheimii*. However, *T. islamabadensis* and *T. pseudoheimii* showed no significant morphological differences from *T. heimii* [21,51,62]. Moreover, the clade contained sequences of *T. heimii* from its type location: India. Therefore, this clade represents only a single species, i.e., *T. heimii*. There are significant morphological differences between *T. pseudointermedius* and *T. heimii*. *Termitomyces heimii* has a white pileus with a blunt and white perforatorium, a thick annulus, and ellipsoid basidiospores measuring 7–8.4 × 5.5–7 μm [62]. Morphologically, *T. pseudointermedius* resembles *T. intermedius* Har. Takah. & Taneyama in sharing a greyish beige, similarly sized and coloured pileus and stipe. However, *T. intermedius* has a white pseudorhiza; sparse to abundant, thin-walled cheilocystidia which are polymorphic and occasionally bicellular; and abundant, thin-walled, clavate pleurocystidia. Moreover, they are highly divergent in their ITS and mtSSU sequences [2,6].

## 4. Discussion

To date, approximately 71 species of *Termitomyces* have been reported from Asia and Africa [19,23,29,63]. Their notable nutritional and medicinal properties make them a sought-after food commodity [15,30]. In China, a total of 20 species of *Termitomyces* have been previously reported. However, *T. cylindricus* S.C. He and *T. albiceps* S.C. He (originally described from Guizhou Province by He in 1986) were initially synonymized with other Chinese species due to morphological similarities but later were revalidated as distinct species [30]. Meanwhile, another two recently described species [6]—*T. tigrinus* S.M. Tang & Raspé and *T. yunnanensis* S.M. Tang & Raspé—were treated as synonyms of *T. intermedius* and *T. cylindricus*, respectively [30]. Additionally, six species—*T. le-testui* (R. Heim) R. Heim, *T. entolomoides* R. Heim, *T. eurrhizus* (Berk.) R. Heim, *T. mammiformis* R.Heim, *T. microcarpus* (Berk & Broome) R. Heim, and *T. tylerianus* Otieno—lacking voucher specimens and molecular data [12,27,29,64,65,,66], have been temporarily excluded from the list of Chinese species. Thus, only 14 species have been confirmed to distribute in China [4,6,16,17,19,30,31,67] (Table 2).

In this study, three newly described species, *T. albus*, *T. apicoannulatus*, and *T. pseudointermedius*, and a species new to China, i.e., *T. pakistanensis*, are reported from China. These species are mainly distributed in the southwestern (e.g., Yunnan Province) and southern (e.g., Guangxi Province) regions of China. Southwestern and southern China have a subtropical to tropical monsoon climate, characterized by year-round warm and humid conditions with abundant rainfall. These provide long-term stable natural conditions for the survival and activity of termites. Simultaneously, the rich variety of vegetation types and complex ecosystems may further promote the differentiation of termite species, thereby driving the co-evolution and diversity accumulation of their symbiotic *Termitomyces* species. Thus, these areas are likely to harbour a high species diversity of *Termitomyces*, suggesting that they may represent important distribution and evolutionary centres for this group of fungi.

## Figures and Tables

**Table 1:** Voucher specimens and GenBank accession numbers for sequences used in phylogenetic analyses. Sequences newly generated in this study are shown in bold.

Taxon	Voucher	Country	GenBank Accessions			Reference
			ITS	mtSSU	nrLSU	
*Asterophora lycoperdoides*	CBS683.82	/	AF357038	AF357110	AF223191	[47]
*A. parasitica*	CBS170.86	/	AF357037	AF357109	AF223190	[47]
*Lyophyllum decastes*	JM87/16	/	AF357059	AF357136	AF042583	[47]
*L. shimeji*	Lc42	/	AF357060	AF357137	AF357078	[47]
*Termitomyces acriumbonatus*	LAH36362	Pakistan	MT179687	/	MT179690	[25]
*T. acriumbonatus*	LAH36345 (holotype)	Pakistan	MT179688	/	MT179689	[25]
*T. albiceps*	HKAS138507 (epitype)	China	PQ807321	/	/	[30]
*T. albiceps*	HTBM1023	China	PQ807302	/	PQ796798	[30]
*T. assamicus*	GUBH20038 (holotype)	India	OQ346313	/	/	[22]
*T. assamicus*	GUBH20039	India	OQ976999	/	/	[22]
*T. aurantiacus*	BR5020070572530	Senegal	OQ275438	OQ275504	OQ275322	GenBank
*T. aurantiacus*	BR5020149963016	Cameroon	OQ275437	OQ275503	OQ275321	GenBank
*T. boluoshanensis*	HKAS145341 (holotype)	China	/	/	PQ796817	[30]
*T. boluoshanensis*	HTBM1049	China	/	/	PQ796820	[30]
*T. bulborhizus*	HMAS84444 (holotype)	China	OQ275467	OQ275528	OQ275379	[28]
*T. clypeatus*	BR5020169447855	Congo	OQ275461	OQ275490	OQ275382	GenBank
*T. clypeatus*	BR5020212704478V	Mali	OQ275460	OQ275489	OQ275381	GenBank
*T. cryptogamus*	P5 (holotype)	South Africa	MW251838	/	MW567773	[48]
*T. dhofarensis*	RAK-22-0025	Oman	OR297696	/	OR338597	[18]
*T. dhofarensis*	NHZ-22-001 (holotype)	Oman	OR297694	/	/	[18]
*T. entolomoides*	BUMR06	India	MK743955	/	/	GenBank
*T. eurrhizus*	KUBOT-KRMK	India	MW479420	/	/	GenBank
*T. eurrhizus*	UOC MAT MT03	Sri Lanka	KP943505	/	/	GenBank
*T. flavus*	YAAS2021081127 (holotype)	Thailand	PP264695	PP264701	PP264704	[26]
*T. floccosus*	MFLU19-1312 (holotype)	Thailand	MT683161	MN701029	MN633305	[16]
*T. fragilis*	HKAS88912 (holotype)	China	KY214475	/	/	[4]
*T. fragilis*	HTBM0902	China	PQ807352	/	PQ796856	[30]
*T. heimii*	RH33	Sri Lanka	OR139836	/	/	[49]
*T. heimii*	PUN10236	India	MK920156	/	/	GenBank
*T. heimii*	AO-DEBCR-6	India	KT459337		KT459338	GenBank
*T. hongpijizong*	HKAS138523 (holotype)	China	/	/	PQ796830	[30]
*T. hongpijizong*	HKAS138845	China	/	/	PQ796831	[30]
*T. intermedius*	GDGM46569	China	MF488971	/	/	[20]
*T. intermedius*	HKAS 117639	China	ON557370	ON557368	ON556485	[6]
*T. intermedius*	YO198 (paratype)	Japan	AB968241	/	/	GenBank
*T. islamabadensis*	LAH36788 (holotype)	Pakistan	MW520178	/	OM100949	[21]
*T. le-testui*	BR5020152014156	Benin	OQ275442	OQ275507	OQ275344	GenBank
*T. mammiformis*	BR5020168982081	Gabon	OQ275440	/	/	GenBank
*T. mammiformis*	BR5020180381527	Burundi	OQ275439	OQ275506	OQ275323	GenBank
*T. medius*	BR5020211012208V	Côte d’Ivoire	OQ275435	OQ275515	OQ275330	GenBank
*T. medius*	BR5020049544742	Benin	OP179299			[6]
*T. microcarpus*	WH13	Sri Lanka	OR139835	/	/	[49]
*T. microcarpus*	UOC KAUNP MK04	Sri Lanka	KP780436	/	/	GenBank
*T. pakistanensis*	S3 (paratype)	Pakistan	OP688123	/	/	[50]
***T. pakistanensis***	**HKAS84603**	**China**	**PX953039**	**/**	**/**	**This study**
*T. pseudoheimii*	CMUB40069	Thailand	PQ897224	PV020679	PQ897223	[51]
*T. pseudoheimii*	SDBR–CMUNKP2013	Thailand	PQ897225	PV020680	PV020680	[51]
*T. radicatus*	MRNo173	Thailand	LC068787	/	/	GenBank
*T. robustus*	BR5020166518664	Benin	OQ275445	OQ275547	OQ275335	GenBank
*T. schimperi*	BR5020149959941	Cameroon	OQ275414	OQ275524	OQ275371	GenBank
*T. schimperi*	BR5020168467755	Rwanda	OQ275413	OQ275523	OQ275370	GenBank
*T. sheikhupurensis*	LAH35710 (holotype)	Pakistan	MT192217	/	MT192228	[5]
*T. sheikhupurensis*	LAH36413	Pakistan	MT192218	/	/	[5]
*T. srilankensis*	FUOR0016 AGS (holotype)	Sri Lanka	ON685313	/	/	[52]
*T. srilankensis*	CUHAM957	India	PP915962	/	/	GenBank
*T. striatus*	TERM006	Thailand	MN160260	/	MN160260	[23]
*T. striatus*	BW18	Philippines	OP179298	/	/	[6]
*T. striatus*	BR5020168468769	Rwanda	OP179297	OP179294	OP168081	[6]
*T. striatus* f. *bibasidiatus*	DM280B	Cameroon	/	KY809193	KY809241	[19]
*T. striatus* f. *subclypeatus*	DM370B	Cameroon	/	KY809220	KY809268	[19]
*T. subclypeatus*	BR5020149965034	Cameroon	OQ275427	OQ275513	OQ275365	GenBank
*T. subumkowaan*	DM260B	Cameroon	/	KY809227	KY809275	[19]
*T. subumkowaan*	BR5020027327701	Burundi	/	OQ785347	OQ753824	GenBank
*T. tigrinus*	HKAS107560 (holotype)	China	MT683156	MT683152	MT679729	[6]
*T. titanicus*	BR5020172891379	Congo	OQ275443	OQ275508	OQ275336	GenBank
*T. titanicus*	AZ1	Zambia	OQ645458		OQ644496	GenBank
*T. tylerianus*	BR5020169473144	Congo	OQ275417	OQ275522	OQ275353	GenBank
*T. upsilocystidiatus*	MFLU19-1289 (holotype)	China	MT683160	MN636642	MN636637	[16]
*T. upsilocystidiatus*	HKAS124533	China	OQ275446	OQ275544	OQ275318	GenBank
*T. yunnanensis*	HKAS124501 (holotype)	China	OP179295	OP179290	OP168083	[6]
***T. pseudointermedius***	**HKAS151727**	**China**	**PX924556**	**PX924566**	**PX916415**	**This study**
***T. albus***	**HKAS151726**	**China**	**PX924552**	**PX924563**	**PX916413**	**This study**
***T. albus***	**HKAS92459**	**China**	**PX924555**	**PX924564**	**PX916414**	**This study**
***T. albus***	**HKAS74497**	**China**	**PX924554**	**/**	**/**	**This study**
***T. albus***	**HKAS73036**	**China**	**PX924553**	**PX924565**	**/**	**This study**
***T. apicoannulatus***	**HKAS151733**	**China**	**PX924559**	**PX924571**	**PX916418**	**This study**
***T. apicoannulatus***	**HKAS151728**	**China**	**PX924557**	**/**	**PX916416**	**This study**
***T. apicoannulatus***	**HKAS151729**	**China**	**PX924558**	**PX924567**	**PX916417**	**This study**
***T. apicoannulatus***	**HKAS151732**	**China**	**PX924562**	**PX924570**	**PX916421**	**This study**
***T. apicoannulatus***	**HKAS151730**	**China**	**PX924560**	**PX924568**	**PX916419**	**This study**
***T. apicoannulatus***	**HKAS151731**	**China**	**PX924561**	**PX924569**	**PX916420**	**This study**

**Table 2:** Information on 14 known species of *Termitomyces* in China.

Taxon	Voucher	References
*T. albiceps*	HKAS138507 (epitype)	[30,68]
*T. albus*	HKAS151726 (holotype)	This study
*T. apicoannulatus*	HKAS151733	This study
*T. boluoshanensis*	HKAS145341 (holotype)	[30]
*T. bulborhizus*	HMAS84444 (holotype)	[28]
*T. cylindricus*	S37	[30,68]
*T. flavus*	YAAS2021081127 (holotype)	[30]
*T. fragilis*	HKAS88912 (holotype)	[4]
*T. heimii*	AO-DEBCR-6	[13]
*T. hongpijizong*	HKAS138523	[30]
*T. intermedius*	GDGM46569	[20]
*T. pakistanensis*	HKAS84603	This study
*T. pseudointermedius*	HKAS151727 (holotype)	This study
*T. upsilocystidiatus*	MFLU19-1289 (holotype)	[16]

## Data Availability

The original contributions presented in this study are included in the article. Further inquiries can be directed to the corresponding author.
